# Assessment of Two Online Interventions for Veterans With Chronic Pain: Protocol for a Randomized Controlled Efficacy Trial

**DOI:** 10.2196/70601

**Published:** 2025-08-13

**Authors:** Erin D Reilly, Hannah L Grigorian, Alicia A Heapy, Bella Etingen, Megan M Kelly, Noah R Wolkowicz, Caitlin M Girouard, Timothy P Hogan, Katarina Bernice, Timothy Bickmore

**Affiliations:** 1 Mental Illness Research, Education, and Clinical Center Veteran Affairs Bedford Healthcare System Department of Veterans Affairs Bedford, MA United States; 2 Department of Psychiatry University of Massachusetts Chan Medical School Worcester, MA United States; 3 Pain Research, Informatics, Multi-morbidities, and Education Center Veterans Affairs Connecticut Healthcare System Department of Veterans Affairs West Haven, CT United States; 4 School of Medicine Yale University New Haven, CT United States; 5 Research and Development Service Dallas VA Medical Center Department of Veteran Affairs Dallas, TX United States; 6 Department of Health Economics, Systems, and Policy Peter O’Donnell Jr. School of Public Health UT Southwestern Medical Center Dallas, TX United States; 7 Mental Illness Research, Education, and Clinical Center VA Connecticut Healthcare System Department of Veteran Affairs West Haven, CT United States; 8 Center for Healthcare Organization and Implementation Research Veterans Affairs Bedford Healthcare System Department of Veterans Affairs Bedford, MA United States; 9 Khoury College of Computer Sciences Northeastern University Boston, MA United States

**Keywords:** chronic pain, randomized controlled trial, acceptance and commitment therapy, embodied conversational agent, digital health intervention, efficacy trial

## Abstract

**Background:**

Chronic pain is a debilitating condition that disproportionately impacts US veterans who manage numerous negative pain-related outcomes. There is an urgent need for accessible, engaging, and innovative treatments that can help veterans with chronic pain better self-manage their pain at home and improve their daily functioning. Technology-delivered acceptance- and mindfulness-based interventions for pain have shown strong efficacy, particularly when they are engaging and tailored to specific client needs. However, more research is needed to assess the impact of such interventions, particularly in terms of pain-related functioning and quality of life.

**Objective:**

The primary aim of this study is to test the efficacy of Veteran Acceptance and Commitment Therapy for Chronic Pain (VACT-CP), an online self-management pain program, compared to an active online control (Online Pain School) for improving pain-related functioning in a 3-site randomized controlled trial. The secondary aim is to explore psychological flexibility as a potential mediator between pain severity and pain-related functioning.

**Methods:**

This study will use a mixed methods approach to examine the efficacy of VACT-CP in a 2-arm, multisite, randomized controlled superiority trial including 200 participants with chronic musculoskeletal pain compared to Online Pain School. Participants will be assigned to 1 of these 2 online interventions. Both interventions will include 7 modules delivered over 7 weeks, with each module lasting approximately 15 minutes. Mixed effects models will be used to analyze the primary hypothesis that participants in the VACT-CP group will have greater improvement in pain-related functioning (Brief Pain Inventory–Interference subscale) than those in the active control group (Online Pain School). The main acceptance and commitment therapy process mediator (ie, Multidimensional Psychological Flexibility Inventory), pain-related functioning outcomes (Brief Pain Inventory–Interference subscale), and quality of life (Veterans RAND 36-Item Health Survey) will be measured at baseline, end of treatment, and 3 and 6 months after treatment. In addition, qualitative exit interviews will be conducted with a random set of 30 VACT-CP users (n=10, 33% per site) to obtain intervention usability, feasibility, and acceptability information.

**Results:**

The recruitment for this study began in January 2025. It is expected to continue through January 2027. Data collection is expected to be completed by June 2027, and primary data analyses are expected to be completed by early 2028.

**Conclusions:**

Online interventions such as VACT-CP and Online Pain School have the potential to expand access to behavioral interventions that improve quality of life and provide nonpharmacological pain treatment options for veterans experiencing chronic pain. However, research on their impact and underlying mechanisms of change is required to support this area of potential at-home programming.

**Trial Registration:**

ClinicalTrials.gov NCT06058624; https://clinicaltrials.gov/study/NCT06058624

**International Registered Report Identifier (IRRID):**

PRR1-10.2196/70601

## Introduction

### Background

Pain has been identified as one of the most frequently presented medical concerns in primary care for veterans [1]. According to the World Health Organization, pain is an “unpleasant sensory and emotional experience associated with, or resembling that associated with, actual or potential tissue damage” [2]. Perhaps the most complicated to treat and devastating type of pain is chronic pain. Partly due to active-duty physical experiences and injuries, chronic pain disproportionately impacts veterans, who are diagnosed at high rates and with a greater rate of severe pain than nonveterans [1,3]. Many areas of daily living and functioning are impacted by the experience of chronic pain. Veterans with chronic pain face numerous functional difficulties, including poor integration into their community [4] and difficulties in social situations and changes in activities of daily life, sleep, and appetite [1,5,6]. Chronic pain has also been associated with numerous negative outcomes in veterans, including increased morbidity and mortality [7], decreased satisfaction with health care [4], and increased health care use [8].

To reduce the impact of pain, pharmacological treatments such as long-term opioid therapy have often been prescribed [9]. Unfortunately, the short-term relief provided by opioids does not translate into long-term effectiveness [10,11]. In fact, there is little evidence of the long-term efficacy of prescription opioids in chronic pain treatment, and they can lead to problematic substance use and death [5,6,12]. This research has led to recent Centers for Disease Control and Prevention and the US Department of Veterans Affairs (VA)/Department of Defense Clinical Practice guidelines de-emphasizing opioids and suggesting nonopioid medications and nonpharmacological interventions, in particular psychological interventions, as first-line treatment [7]. Thus, to adequately address pain, it is imperative to look beyond pharmacological treatments and address not only the physical but also cognitive and affective factors associated with chronic pain.

With more than 20 years of empirical research supporting its efficacy, acceptance and commitment therapy (ACT) encapsulates the strengths of several existing chronic pain treatment approaches for improved client functioning. ACT is composed of 6 major focus areas that provide a framework for clients to re-engage with the present, their values, and the actions that provide personalized meaning or bring psychological flexibility into their lives [13]. By targeting the catastrophizing thought patterns and affective experiences (negative emotions, memories, and urges) that can cause pain severity to negatively impact functioning, ACT can teach the skills necessary for clients to engage in valued behaviors and functional improvement even when facing cognitive and affective barriers [14-16].

Evidence shows that ACT interventions can increase acceptance of chronic pain, with multiple effectiveness studies producing average effect sizes in the medium to large range, with positive impacts on social functioning and decreased pain-related medical visits even 3 years following treatment [17]. As it leverages a transdiagnostic approach to functional improvement, ACT can be particularly useful when working with clients experiencing challenges across multiple comorbid chronic mental health and pain diagnoses [16]. In addition, when veterans are managing both chronic pain and substance use issues in the context of serious life stressors such as the COVID-19 pandemic, greater psychological flexibility can buffer particularly well against negative outcomes in mental health functioning and quality of life [18]. Therefore, ACT, with its specific focus on functional impairment, valued living, and pain acceptance, may be particularly well-suited to improving quality of life and functioning among veterans with chronic pain.

Behavioral treatment for chronic pain can be labor intensive and may not be offered in all health care systems, leading to limited access for patients with chronic pain [19]. Within the VA, clinician-delivered treatment can also be burdensome for patients, as it may require weekly appointments during business hours for 10 to 12 weeks in addition to appointments with prescribers. At the same time, nonpharmacological pain management is more prevalent among VA-connected veterans compared to non–VA-connected veterans [20]. This suggests that veterans could benefit from increased access to empirically supported, nonpharmacological, VA treatments for chronic pain, particularly when that program can also reduce common barriers to care. The rapidly expanding field of online and mobile-based health care services represents an opportunity to increase veterans’ access to empirically supported nonpharmacological chronic pain and mental health treatments [21,22]. As 91% of the US adults have access to internet-connected devices [23], digital health interventions have the potential to reach veterans in their own homes.

Web- and mobile-based therapeutic interventions for chronic pain could thus improve veterans’ access to psychological interventions for chronic pain, including ACT. Online-delivered ACT for chronic pain has been found to increase activity engagement and pain acceptance while decreasing pain-related distress, anxiety, and depression [24]. Specifically, interactive online systems can provide cognitive and behavioral treatment that leads to lower pain-related interference with daily activities, greater physical and emotional functioning, better sleep quality, and increased quality of life [24,25]. However, although online programs are increasing in popularity and are often as efficacious as in-person treatment, sustained engagement and retention of consumers in online treatment continue to lag [23,26]. Consequently, there remains a critical need to improve online treatment options for ACT for chronic pain to create a more veteran-centered (eg, tailored to their reported needs and common comorbid conditions) and engaging experience as well as test the efficacy of such treatments among veterans with chronic pain.

To address difficulties with engagement, online-supported mental health interventions have explored the possibility of using animated characters, also known as embodied conversational agents (ECAs), as personal guides to support behavioral interventions [27]. Studies suggest that the use of ECAs can increase online-treatment motivation and feedback by mimicking human interactions and dialogue, resulting in improved treatment compliance and use, physical functioning (eg, increased physical activity and diet fidelity), and client-goal achievement [28,29]. The use of ECAs within mental health treatment is expanding rapidly, and ECAs are increasingly being considered and adopted for psychotherapeutic cognitive and behavioral interventions with promising results [30].

Given this research base, our interdisciplinary research team created a 7-week, 7-module ECA-delivered guided online program, “Veteran Acceptance and Commitment Therapy for Chronic Pain” (VACT-CP) [31]. VACT-CP is a therapeutic intervention focused on increasing psychological flexibility or an individual’s ability to reconnect with the present moment and cope with the impact of chronic pain on cognitive, emotional, and behavioral functioning. The VACT-CP website was designed iteratively and evaluated through a small pilot feasibility randomized controlled trial (RCT), which suggested that VACT-CP is feasible, usable, and acceptable [32]. In addition, these preliminary results suggested that VACT-CP can also assist veterans with chronic pain in increasing their pain acceptance and activity engagement and improving co-occurring depressive symptoms.

### Objectives

This study will use a multisite, 2-arm RCT design that tests the efficacy of VACT-CP compared to an active online control (Online Pain School). The goal of VACT-CP is to assist veterans with chronic pain to improve their functioning and quality of life by engaging them in pain psychoeducation, ACT exercises (eg, acceptance and mindfulness), and personal goal setting guided by their values. VACT-CP accomplishes this goal by supporting more adaptive coping skill attainment to help manage pain-related symptoms and functional goals aligned with valued life domains such as social relationships, physical health, or careers. This study aims to evaluate (1) the efficacy of VACT-CP to improve pain-related functioning for veterans with chronic pain, (2) the efficacy of VACT-CP for improving quality of life for veterans with chronic pain, and (3) the ACT processes that underly the relationship between pain severity and pain-related functioning among veterans in the VACT-CP group. Our hypotheses are as follows:

1. Veterans who receive VACT-CP, compared to veterans in the active control condition, will report significantly greater reductions in pain interference (Brief Pain Inventory [BPI]–Interference subscale) after treatment and at 3-month and 6-month follow-ups (hypothesis 1).
2. Veterans who receive VACT-CP, compared to those in the active control condition, will report significantly greater increases in quality of life (Veterans RAND 36-Item Health Survey [VR-36]) after treatment and at 3-month and 6-month follow-ups (hypothesis 2).
3. The effect of pain severity (BPI–Pain Severity subscale) on functioning (BPI–Interference subscale) will be mediated by improvements in psychological flexibility (Multidimensional Psychological Flexibility Inventory; MPFI) after treatment and at 3-month (primary time point) and 6-month follow-ups (hypothesis 3).

## Methods

### Ethical Considerations

This study was approved by the VA Central Institutional Review Board (cIRB; 1758411-3; version 2), and the requirement for written informed consent was waived. Amendments will be processed through the local site institutional review board and cIRB according to VA policy. Participants will be compensated US $70 for the baseline assessment, US $30 for the midpoint assessment (week 3), US $80 for the end of treatment assessment (end of week 7), US $50 for the 3-month follow-up assessment, and US $50 for the 6-month follow-up assessment (total possible compensation of US $280 in either condition). Participants completing an exit interview will receive US $20. Compensation will be given immediately after completing each assessment time point, and all compensation will be provided via Amazon gift cards. Data will be deidentified, coded, and stored in secure online VA server spaces and within secure, locked VA facilities. These spaces will be securely maintained and will be accessible only to research staff directly involved in this project.

### Study Trial Design

**Figure 1:**
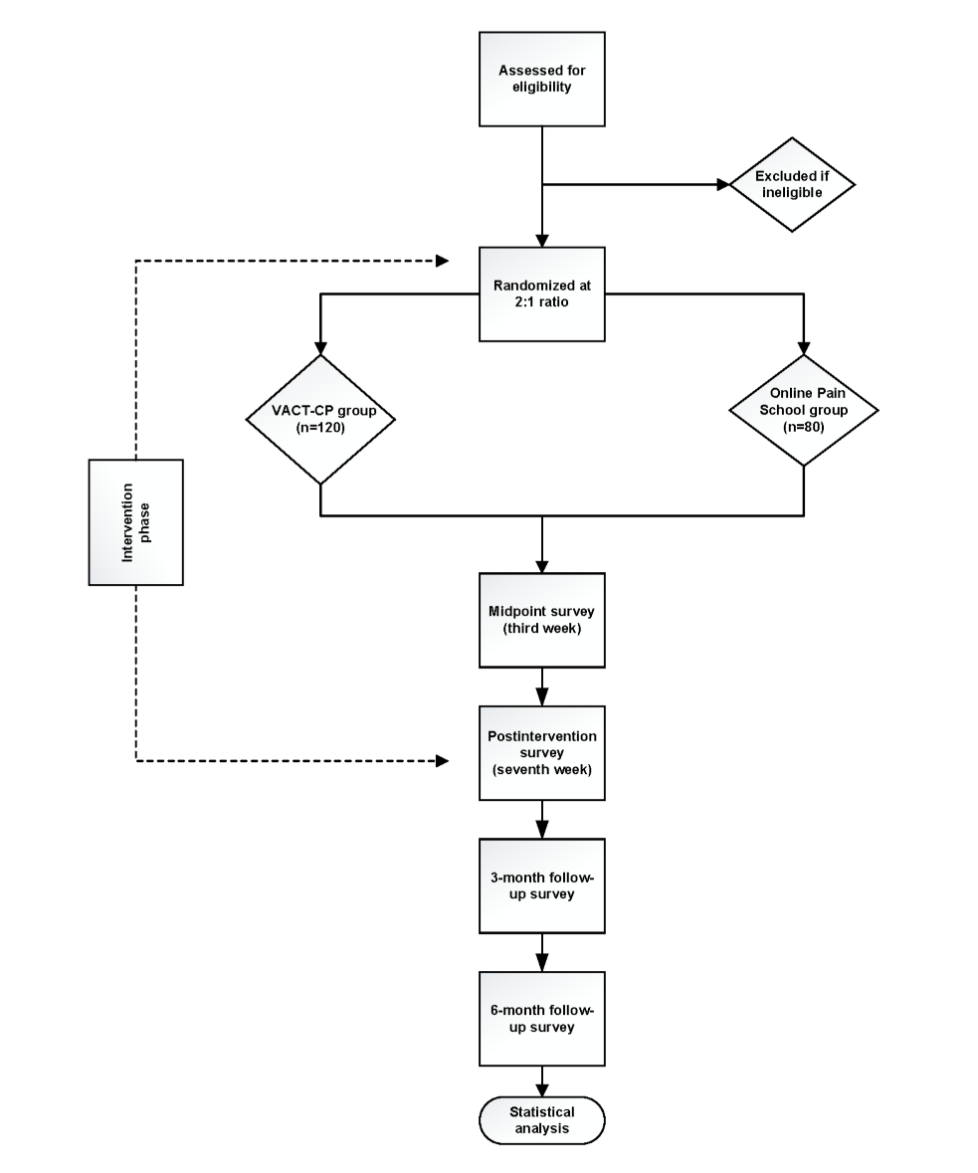
Participant flow diagram. VACT-CP: Veteran Acceptance and Commitment Therapy for Chronic Pain.

This study aims to conduct a 3-site, randomized controlled, parallel group efficacy trial of VACT-CP (n=120) versus an active online control condition (Online Pain School; n=80) with a 1:2 allocation ratio (Online Pain School vs VACT-CP arms) to allow mediation analysis of hypothesis 3 in the VACT-CP group only. Online Pain School is adapted from a common in-person treatment option for veterans, modified for an online format, and delivered through a VA-approved, secure website. All assessment procedures will be delivered remotely via Qualtrics surveys or secure VA teleconferencing system or in person (if requested by participants), including screening, baseline sessions, follow-up surveys (week 3, week 7, 3-month follow-up, and 6-month follow-up), and qualitative interviews (n=30) will assess for VACT-CP program acceptance and gather suggestions for improvement. Participants who discontinue intervention engagement will still be contacted to complete follow-up assessments. The Yale University Data and Safety Monitoring Board will monitor this project; members will be independent from the study sponsor and have no reported conflicts of interest. Reports on recruitment, follow-up, and adverse events will be reported to the Yale University Data and Safety Monitoring Board panel on a quarterly basis. In addition, reporting of adverse events will be provided to the local institutional review board and cIRB committees according to VA policy. Figure 1 presents the study workflow and timeline for each stage of research participation. The SPIRIT (Standard Protocol Items: Recommendations for Interventional Trials) checklist is provided in Multimedia Appendix 1 [33].

### Participant Recruitment

Veterans (N=200) will be recruited and enrolled over the course of 30 months (with a target monthly enrollment rate of 2-3 veterans per site) from 3 US VA medical centers (Bedford, Massachusetts; Dallas, Texas; and West Haven, Connecticut). Recruitment will be conducted through methods such as the distribution of study flyers and information at outreach events and clinics within the VA, mailers sent to veterans identified through medical record reviews, and social media advertisements. We will randomize 200 veterans in a 2:1 ratio, with 120 (60%) veterans assigned to VACT-CP (treatment condition) and 80 (40%) veterans assigned to Online Pain School. Eligibility will be determined based on inclusion and exclusion criteria following a phone screening, medical record review, and abbreviated diagnostic interview during the baseline appointment. The inclusion criteria are (1) current diagnosis of noncancer chronic pain, defined by at least one pain-related diagnosis indicated by an *International Classification of Diseases, 9th Revision* or *International Classification of Diseases, Tenth Revision* code related to either musculoskeletal pain or joint problems or Osteoarthritis and minimum of a grade 1 (mild) or 2 (bothersome) on the Graded Chronic Pain Scale–Revised; (2) possession of a computer and working, high-speed wireless internet connection at home; (3) competency to provide written informed consent; and (4) aged ≥18 years. Individuals with chronic pain who do not meet the inclusion criteria will be provided with resources for VA-delivered pain management care. Exclusion criteria will include (1) any current or lifetime *Diagnostic and Statistical Manual of Mental Disorders, 5th Edition (DSM-5)* psychotic disorder; (2) current or recent diagnosis or treatment of (within 1 month of study entry) *DSM-5* substance use disorder; (3) current use of any other chronic pain–related psychological treatment; (4) clinically significant suicidality within the past year as defined by past-year suicidal behaviors or suicide attempts; and (5) any cognitive or physical impairment that would interfere with study participation or using a computer and providing feedback. Exclusion criteria (1), (2), and (4) will be assessed through baseline interviews (ie, *the Structured Clinical Interview for DSM-5* [34]) and medical record reviews. Participants with additional, comorbid pain diagnoses will not be excluded.

### Study Procedures

Interested study participants will first be screened by phone for preliminary inclusion and exclusion criteria, including veteran status, age, chronic pain diagnostic criteria, and technological requirements for participation. Following the phone screening, participants who remain eligible will have their medical records reviewed for any exclusion criteria. Eligible participants will then be scheduled for a one-on-one baseline appointment during which research staff will obtain verbal informed consent (see Multimedia Appendix 2) and confirm study eligibility by administering relevant modules from the Structured Clinical Interview for DSM-5 to assess psychotic disorders, substance use disorders, or clinically significant suicidality within the past year. Eligible veterans will then complete a battery of self-report measures using a secure online survey platform (eg, Qualtrics). After enrollment, they will be randomized with allocation masked; this process will be completed by an approved third-party research staff member without any contact with participants or involvement in their study activities and assessment. To allow full powering of the planned mediation analysis of the VACT-CP group only, we will randomize in a 2:1 (VACT-CP vs Online Pain School) ratio at the site level with stratification according to pain severity. The principal investigator (PI; EDR) and project coordinator (CMG) will inform participants of their assignment through encrypted emails. Research assistants who manage study assessments will be blinded from knowing a participant’s treatment allocation.

Veterans randomized to the VACT-CP group will receive access to the online program administered as 7 weekly modules (15-20 minutes) programmed and managed by Northeastern University (site PI TB). Online Pain School will serve as an active control condition. Therefore, veterans assigned to this condition will also receive 7 online modules with 15-20 minutes of content based on a common non–ACT-based VA treatment option, Pain School. The adaptation used for this study, Online Pain School, will support veterans in the self-management of chronic pain by providing videos, short exercises, and psychoeducation on different techniques for their “pain management toolbox.” Online Pain School will be equivalent to VACT-CP in terms of frequency and length of modules, number of modules, goal setting, and pain psychoeducation content not related to ACT for chronic pain. All participants will be emailed weekly to remind them to complete the next online module in their respective conditions.

Veterans in both conditions will receive the same follow-up assessments and reimbursements. Participants will be compensated following each assessment time point via gift cards (eg, Amazon gift cards). However, no compensation will be provided for completing modules in either of the website interventions.

### Interventions

#### VACT-CP Online Intervention Condition

VACT-CP is based on previous manualized ACT-CP self-management treatments and workbooks [35-38] and uses ECA technology in the form of “Coach Anne” to create a personalized, therapeutic experience. The 7 VACT-CP online modules are provided as weekly sessions that feature Coach Anne as a virtual treatment guide. All content within the online modules is presented interactively via multimodal conversations with Coach Anne. Coach Anne speaks to the veterans, who respond to her questions using forced-choice text options. Each chosen option triggers unique responses from Coach Anne and routes to different topics or content areas depending on the needs and interests of the veteran user. This process allows personalized and responsive interactions between Coach Anne and the veteran. Veterans are also presented with content to better understand pain management, including videos (eg, veteran narratives and metaphors), in-module assessments (eg, values assessments), and interactive opportunities for goal setting. In addition, the VACT-CP program includes an anytime “Mindfulness Module” and a “My Progress” page, a freestanding module where participants can access their own program data related to their values, goals, and preferred pain management exercises. Veterans will receive a weekly message to remind them to complete their next module each week for the first 7 weeks. After completing the final module, participants will be reminded that they can continue to use the website for the duration of their study involvement, if interested.

#### Online Pain School Active Control Condition

The active intervention control is equated to VACT-CP in terms of treatment duration and number of modules (7 modules over 7 weeks). The content is based on a common non–ACT-based VA pain program option, Pain School, with each module lasting 15 to 20 minutes. The goal will be to provide veterans with more information, tools, and at-home options for pain management. Similar to VACT-CP, each module will include a multimedia format, including videos, text- and image-based self-management information, and questions to assist users in reflecting on their own pain experiences. In addition, similar to VACT-CP, veterans using Online Pain School will receive a message to remind them to complete their next module each week for the first 7 weeks. The online format will also allow the monitoring of website use or “dose.” Pain psychoeducation equivalent to information presented in VACT-CP (eg, chronic pain definitions and activity pacing) will be provided at the same module time point. However, Online Pain School does not include tailoring to the individual veteran’s co-occurring conditions, interactive skills practice, support from the ECA guide Coach Anne, or active components of ACT (eg, mindfulness).

### Measures

#### Demographics and Pain and Mental Health Medication

Demographic measures will include age, gender identity, race and ethnicity, and education. We will also include questions related to concomitant medications currently being used for the management of pain conditions as well as conduct a record review for such information using the VA Computerized Patient Record System (the VA electronic medical record system) or the VA Corporate Data Warehouse (a national repository of patient information across the VA).

#### Pain-Related Functioning

The BPI [39] is a widely used assessment tool for both pain-relevant functioning and pain severity, which is sensitive to change across numerous treatment studies. The pain interference scale (BPI-Interference subscale—primary outcome) consists of 7 items that assess the degree of pain interference with functioning across 7 areas: general activity, mood, walking ability, normal work, relationships, sleep, and enjoyment in life. Items are rated on a scale from 0 to 10 (0=no pain or no interference and 10=most pain or most interference). The Pain Severity Index (BPI–Pain Severity subscale) consists of 4 items used to assess pain severity. The BPI has shown good reliability in previous studies within the BPI–Pain Severity (Cronbach α=0.85) and BPI-Interference (Cronbach α=0.88) subscales [40].

#### Mental Health Measures

We will administer the Patient Health Questionnaire-9 [41] to assess depressive symptoms and the 20-item Posttraumatic Stress Disorder Checklist for *DSM-5* [42] to assess posttraumatic stress disorder symptoms.

#### Quality of Life

The VR-36 [43] is a 36-item measure of health-related quality of life, including physical functioning, role limitations due to physical problems, bodily pain, general health perceptions, vitality, social functioning, role limitations due to emotional problems, and mental health. It is often summarized into physical functioning components and mental functioning components and is one of the most widely used and valid measures of physical and psychological well-being.

#### Psychological Flexibility

The MPFI [44] is a 60-item self-report measure that assesses psychological flexibility, the main mechanism of change proposed in our theoretical model. The MPFI assesses 6 dimensions of flexibility (ie, present moment awareness, self as context, acceptance, contact with values, committed action, and cognitive defusion), 6 dimensions of inflexibility (cognitive fusion, self as context, inaction, the lack of contact with values, the lack of contact with the present moment, and experiential avoidance), and 2 global composite dimensions. The MFPI is sensitive to clinical change, and its subscales have demonstrated excellent internal consistencies across several subpopulations [45].

#### Pain Acceptance

The Chronic Pain Acceptance Questionnaire (CPAQ; [46]) is a 20-item survey measuring willingness and engagement in valued life activities and domains, even during times of pain. Higher CPAQ scores indicate higher activity engagement, pain willingness, and overall pain acceptance. Pain willingness reflects a pattern of refraining from attempts to control or avoid pain, while activity engagement represents the degree of engagement in valued activities irrespective of pain. The CPAQ is a well-validated measure with high internal consistency and test-retest reliability [47].

#### Valued Living

The Chronic Pain Values Inventory [15] is a 12-item self-report measure that measures the agreement between a veteran’s values and their life. Accordance is measured across areas of living, including work, health, and family, and calculated by subtracting an “importance” rating from the “success” rating across each domain. Higher agreement with values is correlated with lower pain-related anxiety, lower overall perceived disability, and greater client functioning. These associations hold even in the context of high levels of pain [15]. Good reliability has been shown in previous studies for accordance scores across domains (Cronbach α=0.82) [32].

#### Intervention Usability

The System Usability Scale [48] is a 10-item measure rated on a 5-point Likert scale that assesses the overall usability of a system. Generally regarded cutoff scores for usability include scores of ≥68 (above average usability) and ≥80 (high usability likely to be recommended).

#### Satisfaction With Treatment

Global treatment satisfaction will be assessed using the Client Satisfaction Questionnaire-8 [49]. This 8-item scale measures the perceived quality and effectiveness of the chronic pain intervention. This scale showed acceptable internal consistency within the previous online VACT-CP pilot study (Cronbach α=0.93) [32].

#### Qualitative Semistructured Exit Interview

A semistructured 30-minute qualitative interview using open-ended questions (Multimedia Appendix 3) will be conducted after the participants complete the intervention to assess acceptability and dissemination issues. A random set of 30 VACT-CP users (n=10, 33% per site) will be identified and invited to participate until the goal of 10 interviews per site is reached. Interview questions will explore participants’ experiences with accessing and using the VACT-CP program online, perceived effects of the treatment’s core components, willingness to recommend the program to others, and any problems or concerns encountered during its use. In addition, we will inquire about past strategies or tools used to address their chronic pain issues and whether the VACT-CP treatment was more useful than previous options or strategies. Finally, participants will be asked to indicate what changes they would make to the treatment to assist in self-management of their chronic pain.

### Sample Size Considerations

The aims of this efficacy study align with the recommendations for stage 2 development of behavioral therapies [50,51]. Therefore, the primary aim of this RCT is to evaluate treatment group–related changes in pain-related functioning and quality of life outcomes. Secondary aims include examining whether the expected mechanism of behavior change (psychological flexibility) mediates the relationship between pain severity and pain-related functioning for veterans receiving VACT-CP. We expect VACT-CP will produce a small to medium treatment effect consistent with previous ACT studies [28].

We will use mixed effects modeling to test for a time by intervention interaction. Our power analysis and sample size estimation is based on our primary outcome, and research suggesting a clinically meaningful desired difference of 1 point on the BPI-Interference [52]. We expect the correlation between time points to be approximately 0.2 based on current preliminary data. To achieve a desired power of 0.80 and a type I error rate of 0.05 to detect a difference of 1 between the (fixed) group means, we estimate that we will need a minimum of 128 participants, with 64 participants per group. To ensure sufficient power for the planned aim 3 mediation analyses (focused on investigating ACT processes within the VACT-CP group), we will over-recruit in the VACT-CP arm to meet the recommended minimum sample size of 120 participants for mediation analysis. We will use path analysis to model the main independent variable X (pain severity), the mediator M (change from baseline to end of treatment in psychological flexibility as measured using the MPFI), and the main outcome variable Y (pain-related functioning) after treatment and at 3-month and 6-month follow-ups. We have thus used a more conservative group size estimate of 80 in the active control group and 120 in the VACT-CP group to fully power both the efficacy evaluation (hypotheses 1 and 2) and the mediational analyses (hypothesis 3).

With an expected attrition rate of 20% based on pilot data, we will obtain consent from up to 240 participants to allow for successful randomization of a total sample of 200 participants. It is possible that attrition will be greater for some clinical groups than for others (eg, veterans with additional symptoms related to a comorbid mental health issue). Therefore, variables of interest may be associated with attrition, and missing data will not be at random, which could then introduce bias in results despite randomization. To address this possibility, we will attempt to determine whether missing data are missing at random or missing completely at random. We will do so by investigating whether baseline measures (depression symptom severity and posttraumatic stress disorder symptom severity) predict attrition and whether statistical testing suggests that data are not missing at random, and we will modify analyses accordingly (eg, via stratification).

### Primary Analyses

Efficacy for the VACT-CP system will be assessed for reductions in pain-related functioning (BPI-Interference subscale; hypothesis 1) and improvement in quality of life (VR-36; hypothesis 2). To test hypothesis 1, which posits that participants’ pain-related functioning (BPI-Interference subscale) will improve more in the VACT-CP group than in the active control group (Online Pain School), 3-month outcome scores will be regressed onto a dichotomous group variable (ie, dummy coded, with Online Pain School group as reference) within a mixed model. The PI will have overall responsibility for monitoring the integrity of the study data and participant safety. All analyses will follow the intention-to-treat approach. We will test hypotheses on repeatedly measured quantitative outcomes using mixed effects models, which will allow for different numbers of observations per participant and use of all available data and will be unaffected by randomly missing data. Models will include a condition-by-time interaction and will adjust for significant or substantive demographic and clinical variables as indicated by preliminary analyses. A significant interaction term in the final model will imply a significant difference in trajectories between the conditions, in which case predicted means or mean frequency of use and rates will be presented separately for each time point. In addition, a significance test of the regression coefficient for the variable identifying group membership will also result in a rejection of the null hypothesis that there is no difference between groups on the BPI-Interference subscale. Hypothesis 2 (that the treatment effect will be similarly present for quality of life; VR-36) will be analyzed using the same regression model. All hypothesis testing will be performed using 2-sided tests with an α of .05 and will use the Benjamini-Hochberg Method to adjust for multiple testing. In addition, exploratory models such as those used to test hypotheses 1 and 2 will be fit to assess outcomes immediately after the treatment and at 6-month follow-up.

To investigate the proposed mechanism of change in VACT-CP (hypothesis 3), we will analyze whether BPI-Interference subscale improvements are mediated by psychological flexibility after treatment and at 3-month and 6-month follow-ups. Similar to previous ACT process mediation procedures [53], structural equation modeling and a bias-corrected bootstrap approach will be used to examine the indirect effect of pain severity on pain-related functioning through pretreatment and posttreatment changes in psychological flexibility. We will model the main independent variable X (pain severity as measured by the BPI-Severity subscale), the mediator M (change from baseline to end of treatment in psychological flexibility as measured by the MPFI), and the main outcome variable Y (pain-related functioning as measured by the BPI-Interference subscale) at 3 months after intervention. A bias-corrected bootstrap procedure with 3000 bootstrap samples will be used to produce a 95% CI around the mediation effect estimate. A 95% CI excluding zero will be interpreted to be statistically significant at a *P*<.05 level. In accordance with previous mediation methods [54], mediation will be assumed demonstrated if paths X and Y and XY (the indirect effect) are statistically significant based on acceptable or adequate model fit. Model fit will be assessed using the chi-square goodness-of-fit statistic and approximate fit indices, including root mean square error of approximation, the goodness-of-fit index, the standardized root mean square residual, and the chi-square/*df*. We will repeat this analysis for posttreatment and 6-month time points as well. This analysis can provide further support for the mechanisms by which the ACT intervention works as well as evaluate the potential longevity of ACT process changes on pain-related functioning.

Additional data will be provided by the Client Satisfaction Questionnaire-8 and semistructured qualitative interviews with VACT-CP users (n=30) as well as by tracking website use information and calls or emails to research staff with concerns. Analysis of the postintervention interviews will be done using notes taken by one of the team members during the exit interview; interviews will also be audio recorded and transcribed. Study staff will apply qualitative analytic techniques to these data (eg, coding for and extracting major themes) to assess both barriers to and facilitators of the use of the VACT-CP system. A modified consensual qualitative research (CQR) approach [55] will be used to code the transcribed videos. CQR can be particularly useful in allowing a team of coders to identify themes arising in both structured and more open-ended interview moments. An initial codebook will be developed using coding based on the key activities and questions in the user testing protocol. These procedures are based, in part, on the successfully piloted interview procedures and script from the initial VACT-CP pilot study. Primary research staff will use the previous pilot codebook in identifying use, usability, and usefulness themes within the interviews. The initial codebook will be used by 2 to 3 coders throughout the data analysis process to foster multiple perspectives until consensus is reached among the coders about the meaning of the data. In addition, at least 1 auditor will check the work of the primary team of coders and serve to minimize potential bias. We will report the frequency of emerging domains and themes. Themes will then be categorized according to the following CQR groupings: general (includes all or all but 1 of the cases), typical (includes half or more of the cases up to the cutoff for general), and variant (at least 2 cases up to the cutoff for typical). We will use these data to discuss broader scientific and implementation-related barriers and facilitators for interventions for patients with chronic pain within the VA system. The themes generated by these data will be used to identify usability issues, perceived usefulness of VACT-CP, and potential areas for revision. The qualitative usability and usefulness information will be triangulated with quantitative data and incorporated into the primary outcome manuscript on VACT-CP efficacy.

## Results

Participant recruitment began in January 2025 and is expected to be completed by January 2027. Data collection is expected to be completed by June 2027, and primary data analyses are expected to be completed by early 2028.

## Discussion

### Potential Applications

A substantial gap remains in the availability of at-home pain self-management treatment options to help veterans increase their functioning and quality of life in the context of their chronic physical, psychological, and social concerns. The VACT-CP program, available at any time and to any veteran with internet and computing technology access, is innovative in its approach and has several potential applications. First, VACT-CP is the only VA-developed and tested website program for chronic pain support and has the potential to improve access to chronic pain treatment for veterans. Further, unlike many online self-management apps and online programs within VA, the VACT-CP treatment uses ACT as an evidence-based alternative to traditionally available and in-person chronic pain treatments (eg, Cognitive Behavioral Therapy for Chronic Pain) to extend treatment targets to pain acceptance and value-based living. Thus, VACT-CP has the potential to add needed emphasis to at-home treatment targets, such as pain-related functional improvement and quality of life. In addition, VACT-CP is uniquely interactive, as the 7-module program is guided by the novel use of an ECA, “Coach Anne,” who can provide user-centered feedback and tailoring through interactive dialogue, assessments, and exercises. The focus on interactive, personalized care has the potential to enhance treatment engagement for veterans. Finally, consistent with VA priorities and clinical guidelines, VACT-CP could improve access to a nonpharmacological pain management treatment option that is readily available to veterans experiencing chronic pain to allow them to gain and sustain a self-care and treatment skill set that applies to both chronic pain and common co-occurring conditions (eg, depression, anxiety, and substance misuse).

### Limitations and Future Directions

If the results of the study are positive, the PI will aim to further study the effectiveness and implementation of VACT-CP nationally through an effectiveness-implementation hybrid type 1 trial to evaluate the implementation context during the planned effectiveness trial. Limitations for this efficacy trial include a lack of more rural VA settings in relation to recruitment activities, inability to include veterans with certain physical impairments (eg, the inability to see and hear the virtual coach), and not including veterans with comorbid substance use disorders, which are common in this population. However, these areas will be expanded upon in future research and revised iterations of the VACT-CP website, should the efficacy trial provide preliminary evidence suggesting further improvement and expansion of this program is warranted. Data from this trial will be disseminated via publications, presentations, and VA-required clinical trials reporting.

### Conclusions

Up to 56% of veterans may be experiencing chronic pain with devastating impacts on their quality of life. Despite this, many veterans may struggle to access pain-related services and, in particular, behavioral interventions for chronic pain. Online interventions have the potential to better fit veteran needs and preferences with in-the-moment content tailoring, asynchronous interactions, and connection to up-to-date online resources for additional pain management exercises and VA resources. This efficacy study of VACT-CP as an online, interactive intervention for ACT for chronic pain could serve to improve veteran care by (1) shifting focus away from pain-related symptoms while emphasizing valued domains of living among veterans with chronic pain, (2) improving quality of life, and (3) improving long-term outcomes associated with pain, such as reduced use of health care services and sick leave.

## Appendix

Multimedia Appendix 1 SPIRIT checklist.

Multimedia Appendix 2 Participant information sheet.

Multimedia Appendix 3 Exit interview protocol.

Multimedia Appendix 4 Peer review report by RRD2 - Musculoskeletal Health & Function, Rehabilitation Research and Development Parent IRG Office of Research & Development (Department of Veterans Affairs, USA).

## Abbreviations

ACT: acceptance and commitment therapy
BPI: Brief Pain Inventory
cIRB: Central Institutional Review Board
CPAQ: Chronic Pain Acceptance Questionnaire
CQR: Consensual Qualitative Research
DSM-5: Diagnostic and Statistical Manual of Mental Disorders, 5th Edition
ECA: embodied conversational agent
MPFI: Multidimensional Psychological Flexibility Inventory
PI: principal investigator
RCT: randomized controlled trial
SPIRIT: Standard Protocol Items: Recommendations for Interventional Trials
VA: US Department of Veterans Affairs
VACT-CP: Veteran Acceptance and Commitment Therapy for Chronic Pain
VR-36: Veterans RAND 36-Item Health Survey

## Data Availability

Data sharing is not applicable to this article as no data sets were generated or analyzed during this study.
