# Supplementary material for: Assessment of Two Online Interventions for Veterans With Chronic Pain: Protocol for a Randomized Controlled Efficacy Trial
Source: JMIR Res Protoc. 2025 Aug 13;14:e70601. doi: 10.2196/70601 (PMC12391840; doi:10.2196/70601)
Supplement: Multimedia Appendix 2 [file resprot_v14i1e70601_app2.pdf]

INFORMATION SHEET FOR "ASSESSING THE EFFICACY OF AN ACCEPTANCE-BASED DIGITAL INTERVENTION TO IMPROVE FUNCTIONING FOR VETERANS WITH CHRONIC PAIN"

You are being asked to participate in a research study conducted by Dr. Erin D. Reilly, funded by a grant from Rehabilitation Research and Development within the Department of Veteran Affairs. You are invited to participate in a research study designed to evaluate two new, online therapeutic interventions to improve the functioning of Veterans with chronic pain. You have been invited because you have chronic pain and are interested in learning new ways to manage it using therapeutic and behavioral techniques and strategies. With this research we hope to learn whether both programs VACT-CP and Online Pain School are helpful in improving the functioning and pain symptoms of Veterans with chronic pain. Your participation in this research study is voluntary. You may choose not to participate or leave the study at any time without penalty or loss of benefits to which you were otherwise entitled.

WHY IS THIS STUDY BEING DONE?

The purpose of this research study is to assess the potential impact and usefulness of two online interventions for pain management, Veteran ACT for Chronic Pain (VACT-CP) and Online Pain School, to evaluate whether and how each online intervention potentially helps Veterans with chronic pain to improve their functioning and pain-related symptoms. This study is being funded by the Rehabilitation R&D within the Department of Veterans Affairs. In addition to online website use, study activities related to completing assessments will take place, depending on your preference, either virtually or at secure research offices located at the VA Bedford Healthcare System (Bedford, MA), VA North Texas Healthcare System (Dallas, TX), or VA Connecticut Healthcare System (West Haven, CT).

WHAT WILL HAPPEN IF I PARTICIPATE IN THIS STUDY?

If you decide to participate in this study, you will be randomly assigned to one of two websites, both of which will provide an online therapeutic intervention to assist with your pain management. This process is like flipping a coin.

For this study, you will have 1 baseline study visit to determine your eligibility for this study. This visit will involve an interview and several questionnaires. You will have the choice to complete this visit and these assessments in person, online, or over the phone. This session will take about 2 hours of your time. If you are eligible for this study, you will be invited to participate in the research study, in either the VACT-CP program or the Online Pain School program. In both programs, you will participate in 7 15min online pain management sessions. For both groups, there will be a midpoint assessment survey (Week 3), which will take approximately 30 minutes. After 7 weeks in the online program, both groups will fill out questionnaires that will take approximately 1 hour. Three and six months later, we will ask you to answer several

VA Central IRB
Effective Date: September 30, 2024

questionnaires again. These follow-up study surveys will take about 45 minutes of your time. You will be given $70 for the first study visit. You will be paid $30 for the midpoint survey. You will also be paid $80 at the end of 7 weeks. You will also receive $50 for the 3-month follow-up survey and another $50 for the 6-month follow-up survey. Thirty participants will also be randomly chosen and given the option to complete a 25-minute qualitative interview, for which they will receive $20. You will be paid by gift-card after each assessment time point.

Intake and Interview Procedures. If you decide to participate in this research study, you will have an in-person or phone screening with a member of the research team and you will be asked to complete questionnaires. We will ask you about your health concerns, pain levels, and technology experiences. You will also be asked questions about possible current and past psychological or emotional difficulties and substance use. This is done because such problems are sometimes associated with pain-related difficulties, and because such problems may affect your efforts to improve your functioning. These interviews and questionnaires will take about 2 hours of your time. We will also ask for the names, contact information, and a release of information for people that you know that would help us be able to get in touch with you. If you do not meet eligibility criteria during this in-person screening, you will be excluded from participation. If you meet eligibility criteria, you will be asked to participate in the initial research phase of the study.

Initial Research Phase. You will be randomly assigned (like a flip of a coin) to one of two groups (VACT-CP or Online Pain School). The reason you will be randomly assigned to a group, rather than you choosing a group is so that researchers can determine the possible usefulness of each online intervention compared to one another. We expect that about 200 people will be randomized in this study.

VACT-CP Group A

If you are assigned to VACT-CP, you will receive access to the online modules, which you will have 7 weeks to complete. Each module will be available to use for a week, and there will be a total of 7 modules you will be asked to complete. The initial module is devoted to an explanation of the treatment rationale, initial psychoeducation on pain-related symptoms, pain interference, and focal concepts of ACT, and assessment of individual pain symptoms. Modules 2-4 will focus on values clarification, acceptance and willingness, mindfulness, with an emphasis on tolerance of pain-related experiences. Modules 5-6 will continue to this focus and incorporate goal-creation and committed action exercises. Module 7 will consolidate and provide feedback on goal-related achievements and will focus on planning for the future. All content will be presented interactively, through text-based conversations with Coach Anne that appear as short videos on the screen. You will hear what Coach Anne “says” and respond to her queries using forced-choice text options that will trigger different responses from Coach Anne as the conversation progresses, to allow the system to responsively interact in a personalized manner with you. During the course of VACT-CP, you will complete the following surveys at five time points: approximately 2 hours for baseline assessments, a total of approximately 2 hours to test the 7-module VACT-CP program and interview over 7 weeks, 30 minutes for the mid-point survey at week 3, approximately 1 hours for the Week 7/post-intervention assessment, and approximately 45 minutes for the 3- and 6- month follow-up surveys. The online modules will take about 15 minutes of your time each week (7 modules total), and you will complete online exercises related to you pain, mood, values, and setting goals for the next week. A research staff member will also briefly check in with you at

VA Central IRB
Effective Date: September 30, 2024

weeks 3 and 6 (approximately 8 minutes) to see if you are experiencing any concerns or issues with the technology that we can assist with.

Online Pain School B

If you are assigned to Online Pain School, you will receive access to the online modules and complete one per week over 7 weeks. “Online Pain School” helps support Veterans’ self-management of their chronic pain, by providing videos, short exercises, and psychoeducation on different techniques for their “pain management toolbox.” The goal is to provide Veterans with more tools and options for pain management, related to their mental and physical health. Modules 1 – 3 will include information on the chronic pain cycle, a review of types or pain, and guidance on managing the functional impacts of pain. Modules 4 and 5 will review pharmacotherapy and chronic pain, as well as issues related to substance misuse and alternatives for healthy pain self-management. Modules 6 and 7 will include support for pain using rehabilitation techniques, flare management suggestions, and considerations to connect to VA resources. During the course of using the Online Pain School website, you will complete the following surveys at five time points: approximately 2 hours for baseline assessments, a total of approximately 2 hours to test the 7-module VACT-CP program and interview over 7 weeks, 30 minutes for the mid-point survey at week 3, approximately 1 hours for the Week 7/post-intervention assessment, and approximately 45 minutes for the 3- and 6- month follow-up surveys. The online modules will take about 15 minutes of your time each week (7 modules total), and you will complete online exercises related to you pain, mood, values, and setting goals for the next week. A research staff member will also briefly check in with you at weeks 3 and 6 (approximately 8 minutes) to see if you are experiencing any concerns or issues with the technology that we can assist with.

Participation in this research study is voluntary. You may refuse to participate and your refusal to participate will involve no penalty or loss of benefits to which you are entitled. You may also discontinue participation at any time without penalty or loss of benefits to which you are entitled. You may withdraw from this study at any time without penalty or loss of VA or other benefits to which you are entitled.

For data already collected prior to the participant’s withdrawal, we will continue to review the data already collected for the study but cannot collect further information, except from public records. You will not be required to answer all survey questions and, if you are interested, you can receive a final report of the aggregated results from the PI (Dr. Erin Reilly) upon request.

ARE THERE ANY RISKS OR DISCOMFORTS?

The intake, mid-point, and follow-up interviews and questionnaires will take time to complete. We estimate it will take approximately 6 hours total over the 8-months to complete study activities and study questionnaires.

You may be uncomfortable answering questions about substance use, emotional and pain-related problems. If you are uncomfortable with any part of the surveys or interview, you may skip the question or take a break. You will have the opportunity to take breaks to minimize fatigue and discomfort. Please let the research staff know if you become too uncomfortable. You can also

VA Central IRB
Effective Date: September 30, 2024

contact the researchers if your symptoms bother you after you go home. If needed, we will contact the psychologist on this study to evaluate you by phone or in person to see if you need any more treatment.

Also, some questionnaires include questions about whether you have had thoughts of harming yourself or others. If the research staff is concerned about your safety during the study, a study clinician may evaluate you and refer you for further evaluation and/or treatment. If a clinician determines that you are a danger to yourself or others, you may be held in a hospital against your will. These actions are to ensure your safety and the safety of others.

At any point during the study, we will discharge you if we are concerned that staying in the study may cause you physical or psychological harm. If the research team discharges you from the study, we will contact your regular clinician to coordinate and provide you with the most Department of Veterans Affairs appropriate care to address these issues. If you do not have a regular clinician, we will discuss possible sources of medical care and encourage you to seek treatment.

ARE THERE ANY BENEFITS?

Your participation may or may not be of benefit to you. The benefits to you include an online care pain care intervention which will teach you skills to help better manage your well-being at home, and careful monitoring during the online treatment. However, we cannot and do not guarantee or promise that you will receive any benefits from this study. If you participation does not benefit you, it will be of benefit to others, as it will contribute to the effort to learn more about the treatment of Veterans with chronic pain.

WHO WILL SEE MY INFORMATION AND HOW WILL IT BE PROTECTED?

The information collected for this study will be kept confidential. Only a code number will identify your research records. The code number will not be based on any information that could be used to identify you (for example, social security number, initials, birth date, etc.) The master list linking names to code numbers will be kept separately from the research data.

All research information collected will be kept in locked files or on VA secure server spaces. VACT-CP website use information will be stored on Northeastern University secure server spaces. Your identity will not be revealed in any reports or publications resulting from this study. There are times when we might have to show your research records to other people.   Only authorized persons will have access to the information gathered in this study. Federal Agencies such as the Office for Human Research Protection (OHRP), Government Accountability Office (GAO) and Food and Drug Administration (FDA) may have access to the records. In addition to the research team, and the VA staff who provide clinical services, other researchers may be granted approval to access this information in the future.

Since we are concerned about your health and safety, there are some situations when we will contact your primary care physician or other clinical professional to provide appropriate care for you, such as to inform him/her that:

- You need to be taken to Urgent Care for medical reasons

VA Central IRB
Effective Date: September 30, 2024

- You report suicidal thoughts or homicidal thoughts
- You are hospitalized
- You experience serious side effects that are a concern to you and/or the study team
- You experience an adverse event or reaction that occurs in the course of the study where the PCP has not already been informed

We have obtained a Certificate of Confidentiality from the Federal Government. This helps protect your privacy by allowing us to refuse to release your name or other information outside of the research study, even by a court order. The Certificate of Confidentiality will not be used to prevent disclosures to local authorities of certain communicable diseases, physical or sexual abuse, child or elder abuse or neglect, or harm or risk of imminent harm to self or others. The Certificate does not protect you if you, someone in your family, or someone you know voluntarily releases information about you.

We will not include information about your study participation in your medical record. In addition to the research team, other researchers may be granted approval to access this information in the future. Federal laws and regulation that protect privacy of medical records will apply to your VA record. A description of this clinical trial will be available on http://www.ClinicalTrials.gov as required by U.S. Law. This website will not include information that can identify you. At most, the website will include a summary of the results. You can search this website at any time.

There are times when we might have to show your records to other people. For example, someone from the Office of Human Research Protections, the Government Accountability Office, the Office of the Inspector General, the VA Office of Research Oversight, the VA Central IRB, our local Research and Development Committee, and other study monitors may look at or copy portions of records that identify you.

WILL I RECEIVE ANY PAYMENT IF I PARTICIPATE IN THIS STUDY?

You will be given $70 for the first study visit. You will be paid $30 for the midpoint survey. You will also be paid $80 at the end of 7 weeks. You will also receive $50 for the 3-month follow-up survey and another $50 for the 6-month follow-up survey. Thirty participants will also be randomly chosen and given the option to complete a 25-minute qualitative interview, for which they will receive $20. You will be paid by gift card within 2 weeks after completing an assessment. If you do not receive your gift card within 2 weeks, please call the Primary Investigator, Dr. Erin Reilly at 781-687-4191.

WHO CAN I TALK TO ABOUT THE STUDY?

In the event of a research related injury, the VA will provide or arrange for necessary medical treatment at no cost to you unless the injury is due to noncompliance with study procedures.

If you should have a medical concern or get hurt or sick as a result of taking part in this study, please contact the Primary Investigator, Dr. Erin Reilly at 781-687-4191.

If you have questions about your rights as a study participant, or you want to make sure this is a valid VA study, you may contact the VA Central Institutional Review Board (IRB) toll free at 1-877-254-3130.

VA Central IRB
Effective Date: September 30, 2024
