# Supplementary material for: Assessment of Two Online Interventions for Veterans With Chronic Pain: Protocol for a Randomized Controlled Efficacy Trial
Source: JMIR Res Protoc. 2025 Aug 13;14:e70601. doi: 10.2196/70601 (PMC12391840; doi:10.2196/70601)
Supplement: Multimedia Appendix 3 [file resprot_v14i1e70601_app3.pdf]

Exit Interview Protocol

Overview

- *First, let's start with the general research study itself. What led you to want to take part in this study to assess an online pain-management website?*

- *How do you feel about technology and its place in your healthcare?*

- *Were you interested in therapeutic interventions for pain online before the study began?*

Coach Anne as an intervention guide:

- What is your overall opinion of the Veteran ACT for Chronic Pain online program?
  - *Was it what you expected?*
  - *How easy was it to use at home?*
  - *Did you complete all the modules? Why or why not?*
  - *How does it compare to other therapeutic interventions you have done for chronic pain?*

- *How do you feel about the program guide, Coach Anne (personality, looks, etc.?)*

Perceived VACT-CP Usability:

- *In general, how did you feel about the 7 Modules that guided you through the ACT for chronic pain program?*

- *Did you use the additional Mindfulness module on the website's main menu? Why or why not?*

- *What feature(s) of the online program did you like most and why?*
  - *How did you feel about the visuals/graphics?*

- *What did you think of the different program options to help you learn more about self-management of chronic pain (use of metaphors, videos, assessments of values, etc.)?*

- *What, if any, exercises or information on the website were **most helpful** to you?*

- *Which exercises or information on the website, if any, did you find l**ess useful?***

VACT-CP At-Home Support for Chronic Pain

- *The VACT-CP program is based on a therapeutic treatment called "Acceptance and Commitment Therapy for Chronic Pain." How did you feel about the acceptance and commitment therapy content related to pain education and management?*
    - *(if necessary, ask about feedback on content related to the pain cycle, primary and secondary or "dirty" pain, pacing vs activity cycling, and the psychological consequences of pain)*

- *Did you think the acceptance and commitment therapy, or ACT, content had an impact on your ability to manage pain, stress, and/or wellness?*
    - *(if necessary, ask about feedback on content related to mindfulness, acceptance, values, setting goals, and/or committed action and willingness)*

- *In what additional ways did the online modules and program benefit you? (mental health, chronic pain, quality of life?)*
    - *If you think about your management of chronic pain or your engagement in valued activities prior to doing the modules to now, have you noticed any changes?*

Suggestions for Improvement

- *What (else) would you suggest to make the website easier to use, or more of interest to Veterans?*

- *Did you have any concerns about using this online program? Do you have remaining concerns after using the online program?*
    - *[possible areas: privacy, data storage, general technology use concerns, etc.]*

- *Would you recommend this program to other Veterans struggling with chronic pain? Why or why not?*
