# Supplementary material for: Assessment of Two Online Interventions for Veterans With Chronic Pain: Protocol for a Randomized Controlled Efficacy Trial
Source: JMIR Res Protoc. 2025 Aug 13;14:e70601. doi: 10.2196/70601 (PMC12391840; doi:10.2196/70601)
Supplement: Multimedia Appendix 4 [file resprot_v14i1e70601_app4.pdf]

SUMMARY STATEMENT

PROGRAM CONTACT: **(Privileged Communication)** ***Release Date:*** **03/23/2023**

Revised Date:

Application Number: **1 I01 RX004804-01**

Principal Investigator

REILLY, ERIN

Applicant Organization: EDITH NOURSE ROGERS MEMORIAL VETERANS HOSPITAL

Review Group: **RRD2**
Musculoskeletal Health & Function

Meeting Date: **02/28/2023** ***RFA/PA:*** **RX22-013**
Council: **MAY 2023**
Requested Start: **10/01/2023**

Project Title: **Assessing the Efficacy of an Acceptance-Based Digital Intervention to Improve Functioning for Veterans with Chronic Pain**
SRG Action: **Impact Score:147 Percentile:7.8**

Human Subjects: **30-Human subjects involved - Certified, no SRG concerns**
Animal Subjects: **10-No live vertebrate animals involved for competing appl.**
Gender: **1A-Both genders, scientifically acceptable**
Minority: **1A-Minorities and non-minorities, scientifically acceptable**
Age: **1A-Children, Adults, Older Adults, scientifically acceptable**
Clinical Research - not NIH-defined Phase III Trial

Project Year	Direct Costs Requested
1	305,426
2	311,694
3	324,657
4	247,038
TOTAL	1,188,815

ADMINISTRATIVE BUDGET NOTE: The budget shown is the requested budget and has not been adjusted to reflect any recommendations made by reviewers. If an award is planned, the costs will be calculated by VA Office of Research and Development (ORD) staff based on the recommendations outlined in the BUDGET COMMENT section and any relevant ORD service-specific limitations.

SUMMARY OF DISCUSSION:
A Subcommittee of the Rehabilitation Research and Development Service Scientific Merit Review Board met in Plenary Session and reviewed the above proposal considering all internal and external reviews. This document summarizes the major points of the discussion concerning the proposed project. In any further development of this project, the investigator should consider carefully all the issues reflected in this Summary of Discussion as well as the more detailed comments in the individual critiques.

GENERAL COMMENTS:
The Subcommittee was unanimous on the following points:

Strengths:
- The applicant proposes a study to test a behavioral intervention to reduce chronic pain, which is a problem that disproportionately affects Veterans and severely impacts numerous domains of functioning, and negatively impacts quality of life.
- The proposal provides a strong overview of the evidence-base regarding non-pharmacologic treatment approaches, including “acceptance commitment therapy” (ACT), which is a therapy directed at increasing psychological flexibility which has previously shown efficacy for chronic pain.
- The proposed research systematically builds upon the work of the Principal Investigator (PI) and her assembled team in which they have modified and translated the ACT program content to an online program. Their preliminary data suggests strong feasibility and usability.
- The proposed study addresses an important problem facing Veterans and has the potential to impact both the delivery of care for Veterans living with chronic pain and to reduce the impact of pain on their daily lives.
- The multi-site RCT in which participants will be randomized to the VACT-CP or an active online program (Online Pain School) provides a robust test of the active intervention ingredients, which is the ACT framework for delivering the pain management program in an online setting with embodied conversational agents.

One Subcommittee member noted several concerns related to the lack of clarity and relevant references for the power analysis section related to Aim 3, which hindered a thorough assessment of the adequacy of the sample size. Also, it might be more appropriate to use a False Discovery Rate approach such as the Benjamini-Hochberg Method to adjust for multiple testing rather than the Bonferroni correction given the number of tests you propose to conduct. A question regarding why pain severity rather than pain interference was included in the mediator analysis was raised. In addition, a notation of the potential lost opportunity of the team to compare the app-based delivery of ACT to a human interventionist (who could still deliver the program virtually and would require an additional control group.

SUGGESTIONS:
None.

COMMENTS ON THE BUDGET:
None.

DESCRIPTION (provided by applicant):
Chronic pain (CP) is a serious concern that disproportionately affects Veterans compared to the general public; Veterans are diagnosed with CP at high rates (47 – 56%) with a 40% greater rate of severe pain than non-Veterans. The negative functional outcomes of chronic pain among Veterans are wide-ranging and include decreased ability to complete daily work activities, less social support from

and closeness with family members, increased chronic health conditions (e.g., heart disease), and higher mortality compared to Veterans without chronic pain. Unfortunately, the use of medication for long-term pain treatment, though often utilized, has both limited efficacy and potentially harmful outcomes. Given these concerns, there is an urgent need for innovative and integrative approaches for non-medical pain self-management. Despite the critical importance of effective pain self-management programs, many Veterans with chronic pain do not engage in the pain self- management programs currently offered by VA. There are numerous reasons for this, including perceived time and transportation concerns and pain-related barriers to attending in-person care. Thus, the development and evaluation of innovative, evidence-based interventions for pain management that can be accessed from home is a crucial step towards improving quality of life for Veterans with chronic pain. One approach with over twenty years of efficacious treatment for chronic pain is Acceptance and Commitment Therapy for Chronic Pain (ACT-CP). ACT is a well-established and VA-approved approach to chronic pain management and focuses on committing to behavior change that reflects personal values, leading to significant improvement in life functioning. Though provided at many VA hospitals, clinician-delivered ACT for chronic pain has not had a nationwide rollout and is not available at all VA pain clinics. Additionally, many Veterans with chronic pain do not access one-on-one therapeutic treatment due to transportation and time issues. Thankfully, for adults with chronic pain outside of the VA, technology-delivered ACT has been found to be acceptable, useful, and efficacious in delivering pain treatment. However, although research suggests it could help with at-home pain management, no ACT for chronic pain online treatment exists specifically for Veterans and their particular care needs. To address this treatment option gap, our research team created an online Veteran ACT for chronic pain (VACT-CP) during the PI's Rehabilitation R&D CDA-2 project. VACT-CP is guided by an interactive virtual coach (Coach Anne) to help address pain-related distress and functional difficulties of chronic pain (e.g., avoidance, reactivity) over seven weeks of treatment. Preliminary findings demonstrate that VACT-CP is highly usable, perceived as helpful, and can help Veterans increase their pain acceptance and pain management. The primary outcomes for this project will be to complete a three-site, fully powered efficacy trial comparing VACT- CP to an active online control condition (total n = 200) to investigate whether VACT-CP can improve pain- related functioning and quality of life. In addition, we will analyze data from the VACT-CP group to assess whether the hypothesized mechanism of change (psychological flexibility) mediates the impact of pain severity on pain-related functioning.

PUBLIC HEALTH RELEVANCE:
Non-pharmacological, accessible chronic pain treatment options are urgently needed for Veterans. This proposed project will provide at-home access to an evidence-based chronic pain treatment using and online acceptance and commitment therapy-based intervention for improving pain-related functioning and quality of life. According to the 2022-2028 Strategic Plan, the VA has also committed to translating and leveraging innovation to inform the development of pain management treatments. Thus, our project will fill a substantial gap in VHA pain services by evaluating a remotely delivered chronic pain treatment, Veteran Acceptance and Commitment Therapy for Chronic Pain (VACT-CP) using a fully powered, multisite efficacy trial (total n=200).

CRITIQUE 1

Significance and Innovation: The proposal cogently outlines the severe impacts that chronic pain have on Veteran's lives across numerous domains and presents information about the current evidence base regarding currently available non-pharmacological treatments. The review indicates that behavioral approaches for chronic pain address cognition and emotional concerns through cognitive-behavioral therapy and more specifically via modifying physical sensations, catastrophic thinking, and

maladaptive behaviors with Acceptance and Commitment Therapy (ACT) having emerged as an efficacious approach. ACT focuses on identifying valued life goals and committing to behavior change using a framework of six major focal areas for patients to address while also teaching necessary skills to help them perform valued behaviors and achieve functional improvement. The innovation of the proposal is using the ACT approach and moving it to be delivered in an online platform using embodied conversational agents (ECAs). The proposed study systematically builds upon the teams work in this area and represents a next logical step. The scientific knowledge to be gained will help advance the scientific knowledge and clinical practice for treating chronic pain in Veterans if the findings yield positive results.

Furthermore, the proposed research builds systematically upon the work of the PI and her assembled team in which they have modified and translated the content of the ACT program for chronic pain to an online program, alpha testing of the first 2 modules and iteratively evaluate feasibility and usability, and then conduct a feasibility RCT pilot of the program to a wait list control group. The PI also has developed and evaluated online ACT intervention programs to address tobacco cessation, mental health, and socio-economically disadvantaged Veterans plus has co-authored a manual on ACT to improve social support for Veterans with PTSD.

Importance and Impact: The proposal addresses an important problem facing Veterans who are significantly more likely to live with chronic and severe pain than non-Veterans. The proposal also is building upon existing strong evidence of non-pharmacological approaches to treat chronic pain and is developing and testing an intervention approach to reduce access to the treatment by Veterans.

Contribution to VHA: The proposed study is likely to yield results that address an important problem facing Veterans and the VA and had the potential to impact the delivery of care and expand the number of Veterans able to receive treatment and experience better health outcomes and better quality of life.

Methods (Data Analysis)**:** This is a multisite, randomized controlled trial with participants randomized to receive the Veterans ACT for Chronic Pain online (VACT-CP, n=120) or an active online control condition (Online Pain School, n=80) which is matched for time, attention, and pain psychoeducation content. The sample size, randomization approach, and interventions are all well conceptualized. The team outlines appropriate plans for recruitment and screening and their analytic plan is appropriate plus accounts for attrition. Their analytic plan is strong and includes using an intent to treat approach for the longitudinal analyses plus examining data regarding intervention feasibility, satisfaction, and usability.

Adequacy of Data: Primary outcomes are Pain-related functioning using the Brief Pain Inventory and Quality of Life (Veterans 36-item Health Survey, VR-36) and the process measure focuses on psychological flexibility using the Multi-dimensional Psychological Flexibility Inventory (MPFI), pain acceptance, and valued living. Secondary outcomes focus on subjective evaluation scores of the interaction with the online program, treatment satisfaction, and conducting a semi-structured interview to assess acceptability and dissemination issues.

Project Organization and Management: Excellent approach for management of the project and study data.

Investigator Qualifications: Strong team of assembled investigators from multiple disciplines.

Dr. Reilly is a clinical psychologist who has used the proposed approach of acceptance and commitment therapy plus has research experience developing and evaluating intervention programs

within this framework and has substantial clinical and research experience addressing chronic pain in Veterans.

Dr. Kelly is also trained as a clinical psychologist and has been involved in conducting interventions using digital approaches to address tobacco use, mental health, and social reintegration.

Dr. Heapy is a clinical psychologist and has expertise in pain management and chronic pain plus has adapted technology for delivery in the digital environment.

Dr. Etingen's background is in Social Psychology and has more than 10 years conducting evaluation projects on health care delivery and outcomes in a VA environment.

Facilities and Resources: Excellent, no concerns.

Human Subjects: No concerns and protections in place.

Critique of Vertebrate Animals Section:

	Yes	No
Research with vertebrate animals?		X

Biohazards and Radioisotopes: Not applicable.

Inclusion of Women, Minorities and Children: No concerns.

Budget (unscored)**:** No concerns.

Data Management and Access Plan (for data sharing, unscored)**:** Present, no concerns.

Overall Strengths: The proposed trial is firmly grounded upon the evidence, builds systematically on previous work in a logical way, is highly innovative, addresses an important problem and can have substantial positive benefits for Veterans, and proposes strong research methodology.

Overall Weaknesses: None to note.

CRITIQUE 2

Significance and Innovation: The proposal addresses an important problem related to the lack of availability of, and access to, high-quality intervention to improve functioning and quality of life for Veterans with chronic pain. In the current rehabilitation regime, Veterans either do not receive any intervention related to their chronic pain, or receive suboptimal amount, in large part due to inadequate supply of trained interventionists; this disproportionally affects Veterans who reside outside of VA locations.

If successful, as the "first step" self-management option consistent with the VA's systematic approach to pain management, the Acceptance-Based Digital Intervention proposed by the project could significantly advance the at-home access to an evidence-based intervention for improving pain-related functioning and quality of life.

Importance and Impact: The proposal states that the effectiveness of ACT interventions is extensively documented; however, their implementation in the emerging digital environments is lacking. Emerging evidence for online ACT for chronic pain does not include considerations for compatibility with VA infrastructure nor with Veteran population. The three proposed interrelated aims are appropriate and are well-poised to advance the goals of the study. Purpose and importance of all aims are clearly stated and well justified.

Aim 1: Insights that inform this development phase are sufficiently justified by previous preliminary study. One weakness may be in the nature of the content provided; Proposal states that Coach Anne has capability to provide “user-centered feedback” and tailoring through interactive dialogue – it is unclear to what extent the content provided is customized based on user data/input, vs. static information.

Aim 2: Comparison with an appropriate control group has sound justification.

Aim 3: Adds a sound theoretical contribution by understanding the mediating effects of the proposed mechanism of change (process of psychological flexibility).

Solid dissemination plan to translate intervention into usable materials for providers and stakeholders.

Contribution to VHA: Proposed research will make a positive difference in the delivery, management or outcomes of VA health services related to care of Veterans with chronic pain. Strong connection to VA initiatives for accessible, nonpharmacological interventions for chronic pain.

Methods (Data Analysis)**:** Hypotheses 1&2: Appropriate use of both quantitative and qualitative data to study the effects of the intervention; appropriate statistical analyses; anticipated co-variates well described.

Hypothesis 3: Informed by approaches used by previous ACT process mediation procedures.

Adequacy of Data: The study is based on a sound, rigorous pilot study that informs major elements of this study, including the content, delivery, and usability of the technology. The depth of the preliminary study is a big strength. Adequate power analysis and sample size determination.

Project Organization and Management: Not applicable.

Investigator Qualifications: Assembled a highly qualified team with demonstrated history of successful collaboration. Extensive domain expertise in ACT (pain interventions) and delivery of online and mobile interventions (tobacco cessation, social support).

Facilities and Resources: Multiple sites support research requirements to enable success of the project. Evidence of institutional support reflecting space, equipment, and resources.

Human Subjects: Ethical procedures followed; evidence of effort to protect participant privacy while obtaining necessary data to conduct study.

Critique of Vertebrate Animals Section:

	Yes	No

Research with vertebrate animals?		X

Biohazards and Radioisotopes: Not applicable.

Inclusion of Women, Minorities and Children: Women and minorities will be included in the study if they meet all study criteria; children excluded due to nature of study and target population.

Budget (unscored)**:** Appropriate; major personnel accounted for, fair participant compensation.

Data Management and Access Plan (for data sharing, unscored)**:** Appropriate.

Overall Strengths: Excellent grantsmanship; clear significance and innovation to the proposed work; excellent team with demonstrated subject matter expertise and history of successful collaboration.

Overall Weaknesses: Minor; there was little description to ascertain the level of interaction afforded by the proposed intervention. In addition to the mechanism of change, the level of interactivity could be a significant mediator of the outcomes.

CRITIQUE 3

Significance and Innovation: No comment.

Importance and Impact: No comment.

Contribution to VHA: No comment.

Methods (Data Analysis)**:**

- The primary aim of this study is to conduct a three-site randomized controlled trial of VACT-CP (n=120) vs. an active online control condition (Online Pain School; n=80), a common treatment option for Veterans adapted to an online format with the assistance of Dr. Tu Ngo (consultant). This will allow for the evaluation of the efficacy of VACT-CP for pain-related functioning (Aim 1) and quality of life (Aim 2) compared to a control matched for time, attention, and pain psychoeducation content, but without ACT mechanisms of change. Similar to past ACT treatment evaluations, participants will be randomized at a 2:1 rate to allow for the minimum required number required for planned mediation analysis (Aim 3). The project will be conducted across four years at the VA Bedford Healthcare System in Bedford, MA, the Hines VA Medical Center in Hines, IL and the VA Connecticut Healthcare System in West Haven, CT. Excellent design and consistent with other similar study.
- They are measuring demographics, functional health, and pain measures. Demographic measures will include age, gender, race and ethnicity, and education. They will also include questions related to concomitant medications currently being used for the management of physical or psychological conditions, as well as conduct a CPRS/CDW record review for such information. To assess for important psychological co-morbidities in chronic pain treatment, they will administer the 9-item Patient Health Questionnaire-9 (PHQ-9) to assess for depressive symptoms, and 20-item PTSD Checklist-5 (PCL-5).
- The primary outcome is pain-related functioning as measured by the Brief Pain Inventory. It is a widely used assessment of both pain-relevant functioning and pain severity which is sensitive to change across numerous treatment studies. The pain severity index (BPI-Pain

Severity) consists of four items used to assess pain severity and the pain interference scale (BPI-Interference) consists of 7 items that assess the degree of pain interference with functioning across 7 areas: general activity, mood, walking ability, normal work, relationships, sleep; and enjoyment in life. Items are rated on a 0–10 (0 = no pain/no interference and 10 = most pain/most interference).

- Quality of Life will be measured by the Veteran's RAND 36 Item Health Survey (VR-36), a 36-item measure of health-related quality of life including physical functioning, role limitations due to physical problems, bodily pain, general health perceptions, vitality, social functioning, role limitations due to emotional problems, and mental health. It is often summarized into physical functioning components (PCI) and mental functioning components (MCI) and is one of the most widely utilized and valid measures of physical and psychological well-being.
- Other measures of pain include The Chronic Pain Acceptance Questionnaire (CPAQ) and the Chronic Pain Values Inventory (CPVI).
- Secondary Outcome Measures include the System Usability Scale (SUS) that assesses human-computer interaction and the Client Satisfaction Questionnaire-8 (CSQ-8) which measures global satisfaction.
- For Power and sample size calculations, they expect the correlation between time points to be approximately 0.2 based on current preliminary data which makes sense. For a desired power of 0.80, a Type I error rate of 0.05 they want to be able to detect a difference of 1 in the BPI-Interference score between the (fixed) group means. While they say this is a clinically meaningful difference, they provide no references. They estimate that they will need a minimum of 128 participants total, with 64 per group. This was not replicable without a Standard Deviation, nor which test they were using for the power calculation.
- In order to be powered to complete Aim 3 planned mediation analyses to investigate ACT processes in the VACT-CP group only, they will over-recruit in the VACT-CP group to meet the minimum suggested sample of 120. They will use path analysis to model the main independent variable X (pain severity), the mediator M (change from baseline to end-of-treatment in psychological flexibility as measured MPFI), and the Y (i.e., BPI-I) at post, 3-, and 6-months. With an expected rate of 20% attrition based on pilot data and will aim to recruit 240 participants to allow for successful randomization of 200 total. This paragraph was difficult to follow and certainly not replicable without knowing the software used or a reference. This implies both treatment and control will be used in the mediator analysis.
- Data analyses will be organized around specific aims and hypotheses; however, in general, they will first perform descriptive statistics and graph the data to better understand its nature and structure. Next, they will perform bivariate analyses (Chi-square tests for categorical variables and Welch's t test for continuous variables) to investigate differences in demographic, pain symptoms, and psychiatric variables between the two conditions to ensure randomization was successful. This is an appropriate way to start analyses rather than jumping right into the complex modeling.
- For the primary longitudinal analyses, they will adopt an intent-to-treat approach and include all available data on subjects who complete at least two timepoints of data and who complete at least one session of the study intervention for each condition. This may cause unwanted variability.
- Because each specific aim will involve investigating the effect of condition and other possible covariates on an outcome over time, they will begin each longitudinal analysis by first testing an unconditional means and an unconditional growth model to ensure enough variance is available to be explained by the predictor and independent variables before model building and hypothesis testing. Again, the right next step.
- Significance level of 0.05 will be used for the primary outcome related hypotheses and

Bonferroni correction will be applied for any post-hoc tests and secondary analyses. All analyses will be performed using SPSS, MPlus, and/or R software. Bonferroni may not be the best approach given the large number of tests to be performed.

- The analysis plan for Aims 1 and 2 is detailed and rigorous and appropriate.
- The analysis plan for Aim 3 looks at each timepoint separately to assess the mediating effect of psychological flexibility on the relationship between pain severity and BPI-interface but doesn't seem to include the treatment effect. This should be explained. Also, they are using an SEM approach to mediation based on sound reference (Woody, E) but it is unclear if this matches the power calculations. Usually, SEM requires large sample sizes.

Adequacy of Data: No comment.

Project Organization and Management:

Investigator Qualifications: No comment.

Facilities and Resources: No comment.

Human Subjects: No comment.

Critique of Vertebrate Animals Section:

	Yes	No
Research with vertebrate animals?		X

Biohazards and Radioisotopes: No comment.

Inclusion of Women, Minorities and Children: No comment.

Budget (unscored)**:** No comment.

Data Management and Access Plan (for data sharing, unscored)**:** No comment.

Overall Strengths: This is a well-designed study with an excellent and detailed analytic plan outside of a few clarifications needed.

Overall Weaknesses: Power and sample size calculations were not replicable and difficult to verify or follow. The team should think about a more robust multiple testing adjustment.

CRITIQUE 4

Significance and Innovation: Chronic pain is a significant problem and is comorbid with anxiety and depression. ACT is a tested therapy to increase psychological flexibility and has been used to treat chronic pain. This is delivered through an app which is tailored to each individual, but also adapted to VA population (examples embedded within the lessons are Veteran specific).

Importance and Impact: Robust testing of behavioral interventions like ACT is essential; delivery in a cost-effective and convenient way would allow greater access to these types of therapies to more people.

Contribution to VHA: Chronic pain is a common source of disability and stress on the Veteran population.

Methods (Data Analysis)**:** Logical app development and promising pilot data are presented. The methodology is detailed and clear. It is apparent that the investigators have drawn from previous experience in the planning for both quantitative and qualitative aspects of the study. They propose the use of reasonable tools to measure both pain and psychological constructs. Control condition is a reasonable match in terms of time and engagement. It appears that those randomized to the control condition will also begin this late, in order to allow a wait-list control condition, although this is not entirely clear. It would be preferable to have at least a small control condition, although the modelling does allow for comparison to baseline.

Adequacy of Data: If data are collected as planned, this will allow testing of the hypotheses outlined in the 3 aims. It seems feasible based on the track record of this group, that enrollment will be able to succeed.

Project Organization and Management: This is a strong investigative team, with adequate experience in these interventions.

Investigator Qualifications: PI is a strong candidate, has utilized spire training to develop the app.

Facilities and Resources: Appropriate.

Human Subjects: Adequate protections.

Critique of Vertebrate Animals Section:

	Yes	No
Research with vertebrate animals?		X

Biohazards and Radioisotopes: Not applicable.

Inclusion of Women, Minorities and Children: Adults only.

Budget (unscored): No comment.

Data Management and Access Plan (for data sharing, unscored)**:** A monitoring entity will assess for safety.

Overall Strengths: The use of ACT, which is well-described, and tailored to both Veterans more generally, and allows tailoring to the individual is a significant and innovative approach to help manage pain. The proposal is well-designed, with clear and attainable aims, methodology well-described and assessment tools likely to measure desired constructs.

Overall Weaknesses: Control group, although allowing for control of engagement, possibly too similar with overlapping content to the intervention. Clarity on the use of a wait-list control (no treatment), which would strengthen the proposal, is needed.

MEETING ROSTER

Musculoskeletal Health & Function
Rehabilitation Research and Development Parent IRG
Office of Research & Development
RRD2
02/28/2023

CHAIRPERSON(S)

CADE, WILLIAM TODD, PHD
PROFESSOR
DOCTOR OF PHYSICAL THERAPY DIVISION
DEPARTMENT OF ORTHOPAEDIC SURGERY
DUKE UNIVERSITY SCHOOL OF MEDICINE
DURHAM, NC 27710

MYKLEBUST, BARBARA M., PHD
BIOMEDICAL RESEARCH CONSULTANT
GERMANTOWN, MD 20875

RUNDELL, SEAN DANIEL, DPT, PHD
ASSOCIATE PROFESSOR
DEPARTEMENT OF REHABILITATION MEDICINE
UNIVERSITY OF WASHINGTON
SEATTLE, WA 98195

MEMBERS

BOST, JAMES E., PHD
ASSOCIATE PROFESSOR
GEORGE WASHINGTON UNIVERSITY
RESEARCH DIVISION CHIEF OF BIOSTATISTICS AND STUDY METHODOLOGY, CHILDREN’S NATIONAL RESEARCH INSTITUTE AT CHILDREN'S NATIONAL MEDICAL CENTER
WASHINGTON, DC 20011

CARBALLIDO-GAMIO, JULIO, PHD *
ASSOCIATE PROFESSOR
DEPARTMENT OF RADIOLOGY
UNIVERSITY OF COLORADO DENVER
ANSCHUTZ MEDICAL CAMPUS
AURORA, CO 80045

CHIMENTI, RUTH LOUISE PORTER, DPT, PHD
ASSISTANT PROFESSOR, DEPARTMENT OF PHYSICAL THERAPY AND REHABILITATION SCIENCE
UNIVERSITY OF IOWA
CARVER COLLEGE OF MEDICINE
IOWA CITY, IA 52242

DREWS, KIMBERLY LEE, MS, PHD
PROFESSOR AND DIRECTOR OF BIOSTATISTICS
LSU PENNINGTON BIOMEDICAL RESEARCH CTR
BATON ROUGE, LA 70808

FROEHLICH-GROBE, KATHERINE
RESEARCH SCIENTIST
CRAIG HOSPITAL
ADJUNCT ASSOCIATE PROFESSOR
SCHOOL OF PUBLIC HEALTH
UTHEALTH SCIENCE CENTER HOUSTON
ENGLEWOOD, CO 80134

GIERISCH, JENNIFER MARIE, PHD, MPH *
RESEARCH SCIENTIST
DURHAM VA MEDICAL CENTER
ASSOCIATE PROFESSOR
DEPARTMENT OF POPULATION HEALTH SCIENCES
DUKE UNIVERSITY SCHOOL OF MEDICINE
DURHAM, NC 27705

GREISING, SARAH M, MS, PHD
ASSOCIATE PROFESSOR
SCHOOL OF KINESIOLOGY
UNIVERSITY OF MINNESOTA
MINNEAPOLIS, MN 55455

HUGHES, ABBEY JEAN, PHD, MA *
ASSISTANT PROFESSOR, ABPP-RP
DEPARTMENT OF PHYSICAL MEDICINE AND REHABILITATION
JOHNS HOPKINS UNIVERSITY SCHOOL OF MEDICINE
BALTIMORE, MD 21287

KAMPER, DEREK, PHD
ASSOCIATE PROFESSOR
JOINT DEPARTMENT OF BIOMEDICAL ENGINEERING
NORTH CAROLINA STATE UNIVERSITY
RALEIGH, NC 27695

LEE, SU JIN *
CLINICAL ASSISTANT PROFESSOR
BYRDINE F. LEWIS COLLEGE OF NURSING AND HEALTH PROFESSIONS
GEORGIA STATE UNIVERSITY
ATLANTA, GA 30303

MORROW, MELISSA M., PHD *
RESEARCH SCIENTIST
MINNEAPOLIS VA HEALTH CARE SYSTEM
PROFESSOR
ENDOWED CHAIR OF NEUROLOGICAL REHABILITATION
UNIVERSITY OF TEXAS MEDICAL BRANCH
GALVESTON, TX 77573

RAMASUNDER, SHALINI, MD
CHIEF, ORTHOPAEDIC SURGERY
DURHAM VA MEDICAL CENTER
ASSISTANT PROFESSOR, ORTHOPAEDICS
DUKE UNIVERSITY
DURHAM, NC 27705

RIEGLER, LINDSAY
INNOVATION SPECIALIST
SPEECH-LANGUAGE PATHOLOGIST/REHABILITATION CARE LI
CINCINNATI VETERANS AFFAIRS MEDICAL CENTER
CINCINNATI, OH 45220

SCHREIBER, KRISTIN, MD, PHD
ASSOCIATE PROFESSOR, HARVARD MEDICAL SCHOOL
VICE CHAIR OF FACULTY DEVELOPMENT
DEPARTMENT OF ANESTHESIOLOGY, PERIOPERATIVE, AND PAIN MEDICINE
BRIGHAM & WOMEN'S HOSPITAL
BOSTON, MA 02115

SESSOMS, PINATA H., PHD
RESEARCH BIOMEDICAL ENGINEER
PHYCORE LAB DIRECTOR
DEPUTY DEPT HEAD
WARFIGHTER PERFORMANCE NAVAL HLTH RESEARCH CENTER
SAN DIEGO, CA 92106

SLAVENS, BROOKE A, PHD, MS *
RESEARCH BIOMEDICAL ENGINEER
CLEMENT J. ZABLOCKI VA MEDICAL CENTER
ASSOCIATE PROFESSOR
MECHANICAL ENGINEERING
UNIVERSITY OF WISCONSIN-MILWAUKEE
MILWAUKEE, WI 53201

WACEK, AMBER, DPT *
RESEARCH PHYSICAL THERAPIST
MINNEAPOLIS VA HEALTH CARE SYSTEM
MINNEAPOLIS, MN 55417

WITHERSPOON, JESSICA, PHD, DPT
ASSOCIATE CENTER DIRECTOR FOR SCIENTIFIC AFFAIRS
BOSTON COOPERATIVE STUDIES
BOSTON VA HEALTHCARE SYSTEM
JAMAICA PLAIN, MA 02130

YAKOVENKO, SERGIY
ASSOCIATE PROFESSOR
DEPARTMENT OF HUMAN PERFORMANCE
WEST VIRGINIA UNIVERSITY SCHOOL OF MEDICINE
MORGANTOWN, WV 26506

SCIENTIFIC REVIEW OFFICER

BRINDLE, TIMOTHY J., PHD, PT
SCIENTIFIC REVIEW OFFICER
DEPARTMENT OF VETERANS AFFAIRS
OFFICE OF RESEARCH AND DEVELOPMENT
REHABILITATION RESEARCH & DEVELOPMENT SERVICE
WASHINGTON, DC 20420

* Temporary Member. For grant applications, temporary members may participate in the entire meeting or may review only selected applications as needed.

Consultants are required to absent themselves from the room during the review of any application if their presence would constitute or appear to constitute a conflict of interest.
